# Modulated effectiveness of rehabilitation motivation by reward strategies combined with tDCS in stroke: study protocol for a randomized controlled trial

**DOI:** 10.3389/fneur.2023.1200741

**Published:** 2023-06-15

**Authors:** Ping Zhou, Wenxi Li, Jingwang Zhao, Siyun Chen, Yufeng Chen, Xia Shen, Dongsheng Xu

**Affiliations:** ^1^Rehabilitation Medicine Research Center, Shanghai Yangzhi Rehabilitation Hospital (Shanghai Sunshine Rehabilitation Center), School of Medicine, Tongji University School of Medicine, Shanghai, China; ^2^Department of Rehabilitation Medicine, Shuguang Hospital, Shanghai University of Traditional Chinese Medicine, Shanghai, China; ^3^Department of Rehabilitation Medicine, Yueyang Hospital of Integrated Traditional Chinese and Western Medicine, Shanghai University of Traditional Chinese Medicine, Shanghai, China; ^4^School of Rehabilitation Science, Shanghai University of Traditional Chinese Medicine, Shanghai, China; ^5^Institute of Rehabilitation Medicine, Shanghai Academy of Traditional Chinese Medicine, Shanghai, China

**Keywords:** stroke, rehabilitation medicine, social cognition, patient-centered care, electric stimulation therapy

## Abstract

**Background:**

Stroke survivors often exhibit low motivation for rehabilitation, hindering their ability to effectively complete rehabilitation training task effectively and participate in daily activities actively. Reward strategies have been identified as an effective method for boosting rehabilitation motivation, but their long-term efficacy remains uncertain. Transcranial direct current stimulation (tDCS) has been recognized as a technique that facilitates plastic changes and functional reorganization of cortical areas. Particularly, tDCS can improve the functional connectivity between brain regions associated with goal-directed behavior when applied to the left dorsolateral prefrontal cortex (dlPFC). Combing reward strategies with tDCS (RStDCS) has been shown to motivate healthy individuals to exert more effort in task performance. However, research exploring the combined and sustained effects of these strategies on rehabilitation motivation in stroke survivors is lacking.

**Methods and design:**

Eighty-seven stroke survivors with low motivation and upper extremity dysfunction will be randomized to receive either conventional treatment, RS treatment, or RStDCS treatment. The RStDCS group will receive reward strategies combined with anodal tDCS stimulation of the left dlPFC. The RS group will receive reward strategies combined with sham stimulation. The conventional group will receive conventional treatment combined with sham stimulation. tDCS stimulation is performed over 3 weeks of hospitalization, 20 min/time, five times a week. Reward strategies refers to personalized active exercise programs for patients during hospitalization and at home. Patients can voluntarily choose tasks for active exercise and self-report to the therapist so as to punch a card for points and exchange gifts. The conventional group will receive home rehabilitation instructions prior to discharge. Rehabilitation motivation, measured using RMS. RMS, FMA, FIM, and ICF activity and social engagement scale will be compared at baseline, 3 weeks, 6 weeks, and 3 months post-enrollment to evaluate patients’ multifaceted health condition based on the ICF framework.

**Discussion:**

This study integrates knowledge from social cognitive science, economic behavioral science, and other relevant fields. We utilize straightforward and feasible reward strategies, combined with neuromodulation technology, to jointly improve patients’ rehabilitation motivation. Behavioral observations and various assessment tools will be used to monitor patients’ rehabilitation motivation and multifaceted health condition according to the ICF framework. The aim is to provide a preliminary exploration path for professionals to develop comprehensive strategies for improving patient rehabilitation motivation and facilitating a complete “hospital—home—society” rehabilitation process.

**Clinical trial registration:**

https://www.chictr.org.cn/showproj.aspx?proj=182589, ChiCTR2300069068

## 1. Introduction

Half of stroke survivors experiences upper limb (UL) functional impairment, which significantly disrupts daily life (1). However, recovery of upper limb function can continue over an extended period with possibility of improvement even after 6 months post-stroke (2). Evidence suggests a positive correlation between therapy dosage and functional recovery (3), emphasizing the importance of encouraging patients to engage in longer and more frequent sessions (4). Yet, the shortage of rehabilitation resources and high cost of medical treatment mean that most patients do not receive adequate supervised therapy during their hospital stay, let alone after returning home (5). Enhancing patients’ motivation for rehabilitation is crucial to maximize the efficiency of rehabilitation training and utilization of rehabilitation services (6). Thus, researchers are increasingly studying how to motivate patients to actively engage in unsupervised rehabilitation exercises both in hospital and at home (7).

Post-stroke apathy is a significant contributor to low motivation in stroke patients (8, 9). Affecting approximately one-third of stroke patients, apathy symptoms can emerge as early as 4 days post-stroke and persist in most patients up to 1 year after the event, with only a small proportion improving over time (10, 11). Apathy manifests as a gradual decline in rehabilitation motivation with a corresponding decrease or refusal to participate in exercises, thereby reducing the exercise dosage (12, 13). If a patient’s rehabilitation motivation remains low, even the most robust medical resources may prove ineffective. On the contrary, when the rehabilitation motivation is boosted, patients can make constructive self-judgment, actively engage in rehabilitation training, face challenge confidently, and enhance motor relearning (14–17).

Six months post-stroke, apathy may not only psychogenic, driven by psychological and social factors, but also organic, resulting from neurobiological changes (18). The possible neural mechanism underlying this apathy is impairment of the brain network related to goal directed behavior (GDB) (19), which includes the anterior cingulate cortex (ACC) and the nucleus accumbens (NAcc) (9, 20). Another plausible explanation is that infarction may cause functional disruption in the connective area following an acute stroke (21).

Current strategies to enhance patients’ motivation primarily aim to boost decision-making abilities and motor relearning capacity. Approaches to promote the decision-making include motivational interviewing (22, 23), integrating individualized rehabilitation programs based on goal orientation and self-management (24–27) and interventions developed from behavioral economics (28, 29). These methods are based on social cognition theory (30) and self-determination theory (31). Education, coaching, and co-setting goals can enhance patients’ self-efficacy, fostering internal motivation for recovery (32).

Methods to bolster motivation and thus motor relearning include sensor technology (33, 34), robot-assisted technology, and virtual reality equipment (35). These methods promote motor motivation by providing refined feedback. As an intrinsic reward, it enhance patients’ ability to analyze and process feedback, improving motor accuracy, proficiency, speed, and coordination (36). However, it is important to note that stroke survivors’ brain exhibit reduced activation of reward systems associated with motivation and motor learning compared to healthy individuals. Thus, even with these strategies, the effect on motivation improvement may not be significant (37). More potent stimulation or treatment may be needed to normalize reward processing (38). Non-invasive neuromodulation techniques like transcranial direct current stimulation (tDCS) and transcranial magnetic stimulation (TMS) can normalize the activation of motivation-related brain regions in stroke patients (37, 39). Some evidence suggests that TMS or tDCS can alleviate apathy and alter the balance of activity in prefrontal and subcortical areas to encourage successful self-regulation in patients (40, 41).

Despite the existence of various interventions to stimulate patients’ motivation toward recovery, improvements are necessary. First, most approaches focus only on patients’ motivation during treatment, with little attention given to the maintenance of motivation post-treatment (42). Yet, the maintenance of high motivation is most critical for patients with chronic disease, as it directly influences their propensity to initiate unsupervised exercise. Second, the cost-effectiveness, healthcare resource consumption, treatment frequency, availability, acceptability, and generalizability of all current motivational interventions need to be examined and enhanced. Third, there is a lack of interventions targeting secondary alterations in stroke, such as functional decline in brain areas associated with motivation and the associated dysfunctional brain network connections. Future efforts should consider combining motivational interventions from multidisciplinary sources. Last but not least, a comprehensive approach to assessing motivation in stroke patients is lacking. A standardized process for evaluating rehabilitation motivation should be established.

Given this content, our team has designed a simple and resource-efficient treatment plan involving a reward strategy combined with a neuromodulation treatment. We aim to modulate patients’ brain neural activity via tDCS, enhancing the functional connectivity of brain regions linked to goal-directed behavior and the reward system. In addition, we plan to collaborate with patients to set treatment goals and offer external rewards to acknowledge their autonomy and self-drive in completing exercises. This approach aims to stimulate their intrinsic motivation and maintain the initiative of the rehabilitation program throughout the “hospital, home, and society” continuum.

### 1.1. Objectives

This randomized controlled trial intends to evaluate the effectiveness of reward strategies combined with tDCS (RStDCS) in improving rehabilitation motivation in 87 patients with chronic stroke (onset over 6 months), compared to RS group without tDCS and conventional treatment group. Through this pilot study, we aim to verify whether reward strategies designed to boost rehabilitation motivation can provide a feasible approach throughout the entire “hospital, home and society” rehabilitation process. The primary objective is to compare the scores of rehabilitation motivation scale and the frequency of patients participating in unsupervised exercises at baseline, 3 weeks, 6 weeks after treatment, and at a 3-month follow-up. The secondary objective is to evaluate the structure and function, activities, and participation of patients within the framework of ICF to observe whether reward strategies can improve rehabilitation motivation, motor ability, daily living ability, and quality of life through self-reporting and assessment instruments.

## 2. Methods and analysis

### 2.1. Study design

The reward strategy combined with tDCS treatment (RStDCS) for improvement of rehabilitation motivation after stroke study is a randomized, single blind, controlled clinical trial. Subjects will be randomly assigned to either the RStDCS group, the reward strategy with sham tDCS treatment group (RS group), or the conventional treatment with sham tDCS treatment (Control group). All patients will receive either true or sham tDCS stimulation and will be provided with an exercise program manual (content varies based on different requirements). Evaluators and statisticians will remain blind to the subjects’ group assignments. Assessments will be conducted at 0, 3, and 6 weeks and followed up at 3 months using assessment scales and behavioral observations. Prior to the assessment, the evaluator will be trained. Assessment scales include the Rehabilitation Motivation Scale (RMS), Fugl-Meyer Assessment Scale (FMA), Function Independent Measure (FIM), and ICF Activity and Participation Scale (ICF). Table 1 shows the schedule of enrollment, interventions, and assessments.

**Table 1:** The schedule of enrolment, interventions, and assessments.

	Study period				
	Enrolment	Allocation	Post-allocation		Close-out
Timepoint**	*−1 day*	0 day	*3 weeks*	*6 weeks*	*3 months*
Enrolment:					
Eligibility screen	X				
Informed consent	X				
Allocation		X			
Interventions:					
Group A (RStDCS)			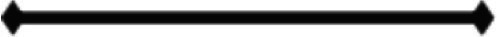		
Group B (RS + sham)			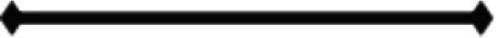		
Group C (Conventional + sham)			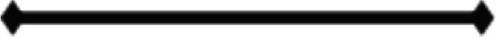		
Assessments:					
RMS		X	X	X	X
FMAS		X	X	X	X
FIM		X	X	X	X
ICF		X	X	X	X

### 2.2. Study population and recruitment

**Figure 1:**
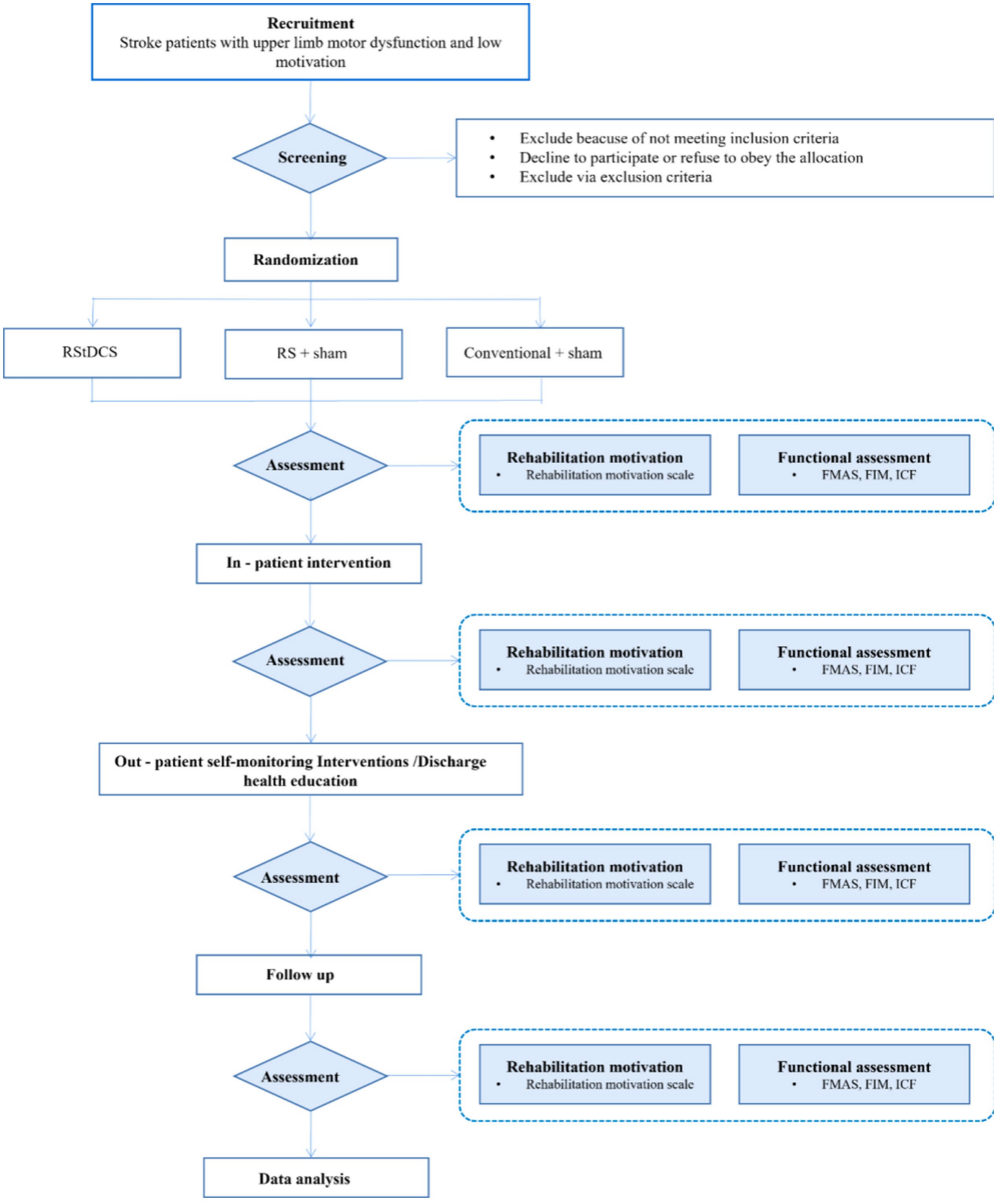
Flowchart of study design.

Initial steps are described in Figure 1. Participants (total of 87) will be recruited from the inpatient and outpatient departments of the Rehabilitation Medicine Center of Yueyang Hospital of Integrated Traditional Chinese and Western Medicine affiliated to Shanghai University of Traditional Chinese Medicine. Patients with confirmed upper limb or hand dysfunction after stroke will be screened for eligibility to participate in this study based on the criteria. Once a patient has been assessed as eligible by the rehabilitation physician, they will receive initial study information. After a minimum 2 weeks of consideration, patients are invited to meet with the research physician to discuss any remaining questions and sign the informed consent.

### 2.3. Inclusion and exclusion criteria

Eligible patients should meet the following criteria:

1. Able to provide written informed consent and understand study’s content, follow-up requirements and are willing to complete questionnaires and provide the necessary information at follow-up visits.
2. Diagnosed with stroke based on the diagnostic criteria for ischemic stroke set by the National Cerebrovascular Academic Conference in 2014.
3. Age 18–70 years, no gender limitation, right-handed.
4. No cognitive impairment or communication impairment, Mini-Mental State Test (MMSE) score of ≥ 27 (43).
5. First stroke onset with the course of disease lasting more than 6 months，less than 3 years.
6. Upper limb and hand dysfunction, Brunnstrom Stage ranged from I to VI.
7. Patients’ rehabilitation motivation scale score of ≤25.

The exclusion criteria of this study are:

1. Serious systemic diseases such as cardiopulmonary diseases that would prevent them from tolerating rehabilitation treatment.
2. (History of) Psychosis, major depression (including suicidal tendencies) or epilepsy, HAMD−17 scores>24 (44).
3. Presence of severe systemic diseases such as diabetes and uremia.
4. Severe joint contracture.
5. Any form of consciousness disturbance (somnolence, confusion, stupor, coma, and delirium, etc.) due to any cause, Glasgow coma score < 15 (45).
6. Contraindications for tDCS according to safety guidelines, such as implanted metal foreign bodies or other electronic devices.
7. Auditory or visual impairment may affect assessment and treatment.
8. Use of drugs that alter the excitability of the cerebral cortex (like antiepileptic drugs, sedatives, and hypnotics, etc.).
9. Severe pain, sleep disturbance, or mental disorder.

### 2.4. Randomization and allocation

Patients are randomized into three groups using a random number table, with allocation via sequentially numbered, opaque, sealed envelopes. Upon determining the eligibility of the subjects, the envelopes are opened in sequence and the subjects are assigned to the corresponding test group.

The researcher generating and preserving the random sequences is separated from the trial and will have no clinical involvement. Neither the subjects nor their routine care therapists will be aware of the group assignments.

### 2.5. Interventions

**Figure 2:**
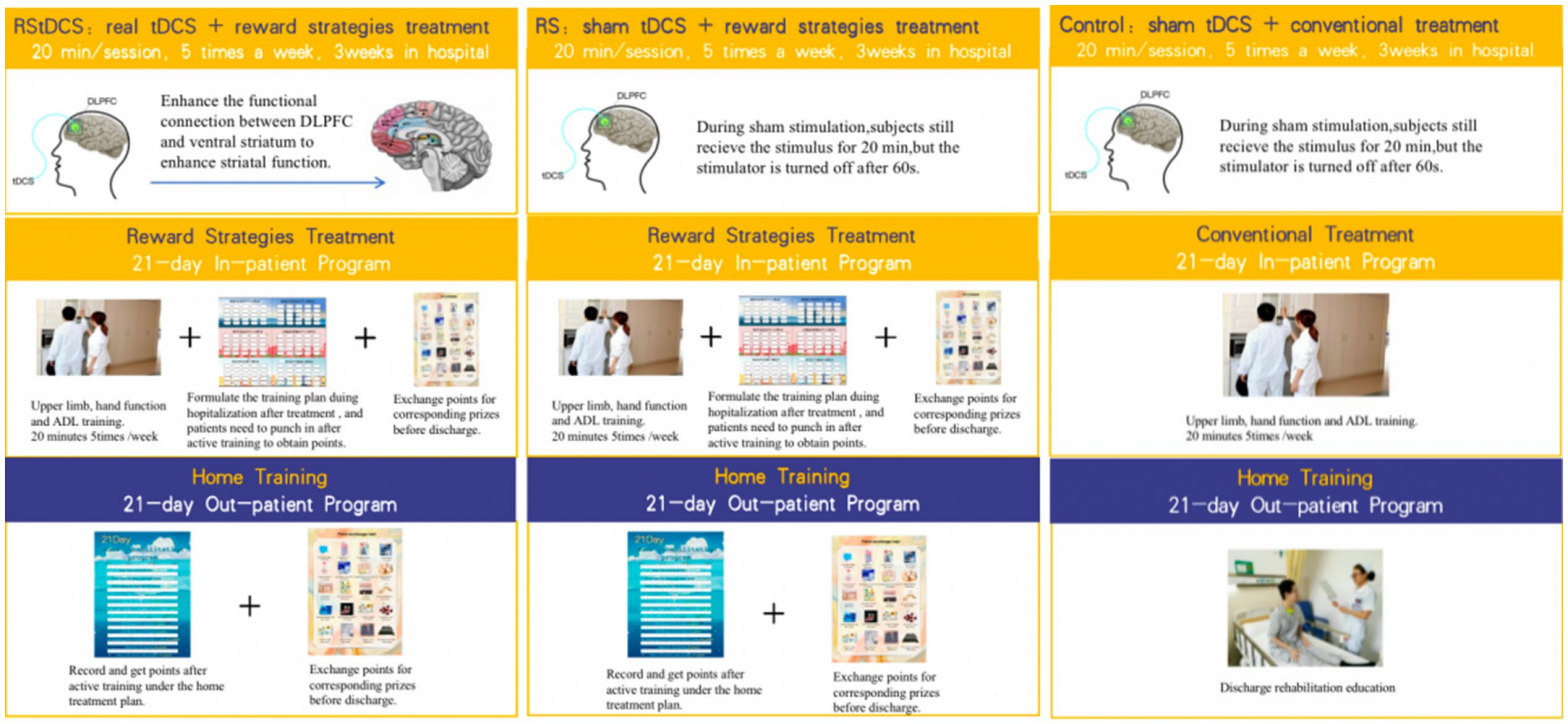
Groups overview and research implementation process.

The trial’s structure and implementation process are depicted in Figure 2. The RStDCS group undergoes upper limb rehabilitation training, complemented with reward strategies and non-invasive neuromodulation techniques. This aims to stimulate patients’ intrinsic motivation through tDCS stimulation of the left dorsolateral prefrontal cortex (dlPFC), an area implicated in predicting successful effort extension. This enables the dorsal anterior cingulate cortex (dACC) to balance potential benefits against required work (46). All the electrodes are placed in saline soaked sponge (5 × 7 cm^2^). The anodal electrode is positioned over left dlPFC (between F3 and F7) according to the International 10–20 system, with cathodal electrode contralaterally on right shoulders. Anodal stimulation is administered at a continuous current of 1.5 mA (0.043 mA/cm^2^ current density over 35 cm^2^ electrodes) for 20 min daily over 3 weeks, totaling 15 sessions. The tDCS stimulation consisted of fade-in and fade-out periods of 30 s to minimize discomfort.

In contrast, the RS group undergoes upper extremity rehabilitation with reward strategies and sham tDCS stimulation, identical in position to the RStDCS group. The current is ramped up and then immediately ramped down in the sham condition in 60 s to mimic the perception of current onset.

**Figure 3:**
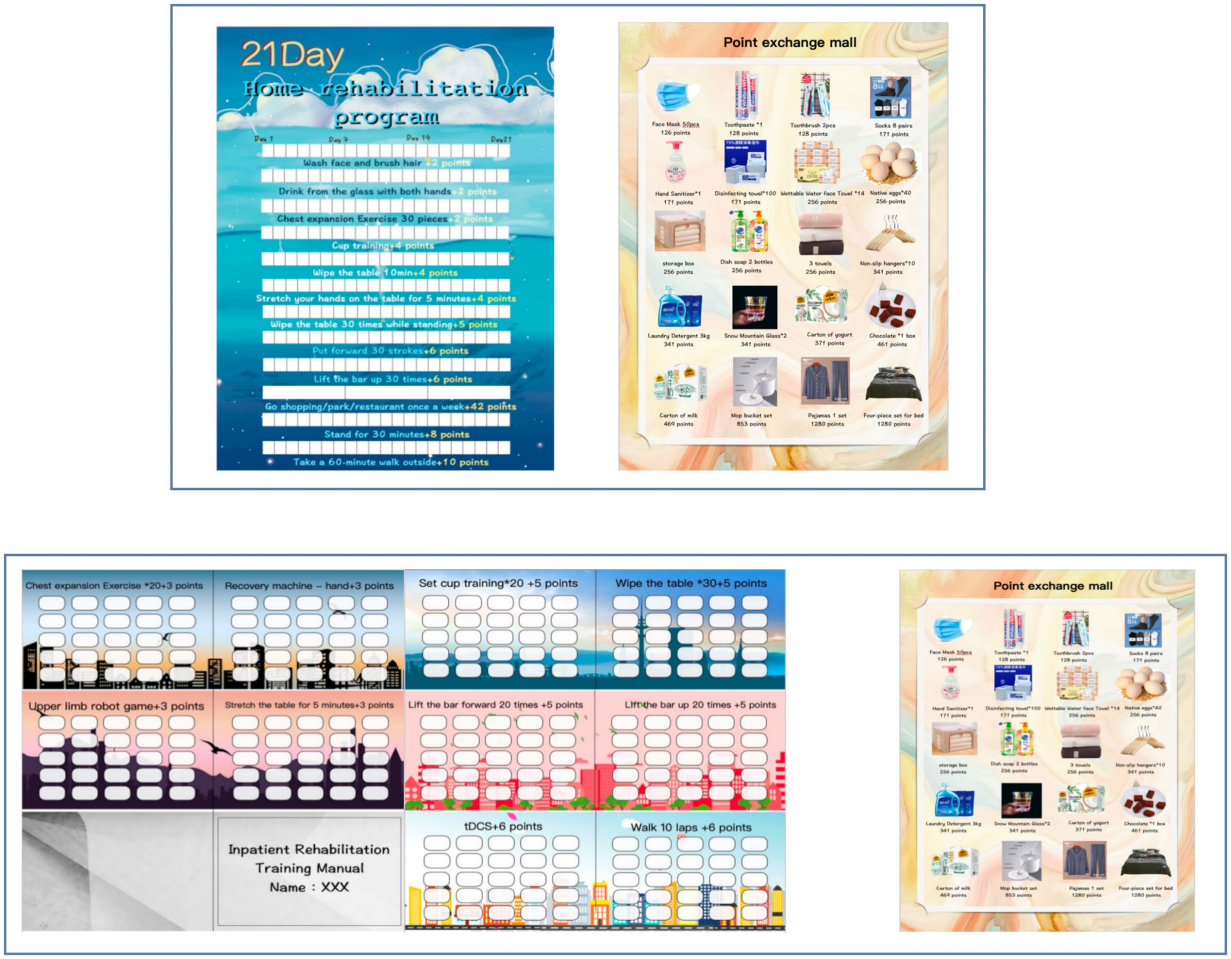
Schematic diagram of reward strategy self-monitor brochure in in-patient and out-patient settings.

Reward strategies involve offering extrinsic rewards in recognition of patients’ independent performance encouraging sustained active rehabilitation motivation. Specifically, a 21-day inpatient-training manual is developed for chronic stroke patient with upper limbs dysfunction and low motivation. Upon discharge, a 21-day home training program is customized for each patient. Figure 3 shows the schematic diagram of reward strategy self-monitor brochure in in-patient and out-patient settings. Both program are tailored according to the Brunnstrom stage of upper limbs and hands, passive range of motion (PROM), muscle tone, muscle strength, fine motor abilities, grip strength, and activities of daily living (ADL). They incorporate exercises to improve and maintain joint mobility, therapeutic exercises, functional training, and ADL training. The Supplementary File 3 shows the contents of the library of active exercise program.

During their 21-day hospital stay, patients in the RStDCS and RS groups undertake the program following their 20-min tDCS stimulation; patients implement the program through self-report and free choice. Therapists award points based on daily task completion. At home without supervision, patients select their task training and self-record their progress to foster intrinsic motivation. At the program’s conclusion, points are tailed and redeemable for gifts (maximum available points can be 2,142, which can exchange gifts worth 267 RMB).

The control group receives conventional upper limb rehabilitation training and sham tDCS stimulation without rewarding strategies. They undergo 20-min training sessions, 5 days a week for 3 weeks. Post-discharge rehabilitation education is provided.

### 2.6. Outcomes measures

Patients are evaluated at baseline, 3 and 6 weeks and at a 3-month follow-up using assessment scales and behavioral observations. Basic data (gender, age, disease duration, affected side, etc.) are extracted from electronic medical records. All data are entered into the paper Case Report Form (CRF) and uploaded to a protected research server online. The assessors are trained prior to data collection. Assessment scales include Rehabilitation Motivation Scale (RMS), Fugl-Meyer Assessment Scale (FMA), Function Independent Measure (FIM), ICF Activity, and Participation Scale (ICF).

#### 2.6.1. Primary outcome measures

The RMS, designed by Litman in 1961 to observe the motivation of patients to participate in physical therapy. Later, it was adapted into a Chinese version by Professor Naiwen Guo’s team. This examiner-rating scale has demonstrated a significant interrater reliability of 0.648 (*p* < 0.001) (22). The content includes complaining level, cooperation level, avoidance, seeking encouragement, pain complaining level, seeking resources, effort level, and attitude on rehabilitation. There are eight questions in total, which are scored by Likert four-point scoring method. Q1, Q4, Q6, and Q8 are positive questions, Q2, Q3, Q5, and Q7 are negative questions, and the score of negative questions are calculated by five minus chosen points. The minimum score of this scale is 8 and the maximum is 32. A score of more than 25 is considered as high motivation. The content of the evaluation scale is in the Supplementary File 1.

#### 2.6.2. Secondary outcome measures

The FMA, created by Swedish doctor Fugl-Meyer on the basis of Brunnstrom rating Scale in 1980. It is a widely recognized and frequently used, with proven reliability and validity (47). FMA can help to monitor and measure the level of ICF structure and function in patients. The FIM, proposed by researchers from the Functional Assessment Research Center of New York State in 1987, assesses the ADL ability of stroke patients comprehensively and objectively (48). The ICF Activity and Participation Scale appraises the health status and health-related condition over the past 30 days covering six areas: understanding and communication, physical activity, self-care, interpersonal interactions, life activity, and social participation. Both FIM and ICF Activity and Participation can measure the level of ICF Activity and Participation level.

Behavioral observation data included the number of therapist-supervised active exercise sessions during hospitalization and the number of self-reported active exercise sessions by patients at home. After 42 days of reward training, the researchers collect the program manual and tally the number of completed sessions.

### 2.7. Data management

Data from patients are gathered using CRFs that comply with the Good Clinical Practice (GCP) standards. Researchers store the assessment results on a protected research server and routinely back up the research folders every 3 months. Informed consent and CRF paper forms are stored in a locked room in the institute’s office. All paper raw data and analysis procedures will be archived to enable future researchers to reproduce or reuse the experimental data.

In data storage, patient study results are recorded using a serial number as a marker. The key to the serial numbers is held by the principal investigator. Only authorized researchers can access this key during the study period, and no participant information will be made public.

### 2.8. Statistics

#### 2.8.1. Sample size

Sample size estimation was made assuming a medium effect size (0.25) seen in previous similar studies (49, 50). G*power software (3.1.9) is used with the means of ANOVA Repeated measures, within-between interaction. The type of power analysis is *a priori*: compute required sample size-given α, power, and effect size. The calculation indicated a sample size of 72 subjects with a desired power of 0.90 at a 5% significance (two-tailed), correlation coefficient of 0.5, and nonsphericity correction of 0.5. Factoring in a 15% dropout rate, we estimated that each group should comprise 29 subjects, making a total of 87 subjects across the three groups.

#### 2.8.2. Statistical analyses

SPSS 26.0 version will be used for data analysis. Chi-square tests are used to compare count data, and descriptive statistical analyses is conducted to study the basic information of subjects in RStDCS group, RS group, and control group. Independent sample *t* tests will be used for intergroup comparisons of measurement data following a normal distribution, while paired sample *t* tests will be used for intragroup comparisons, represented as “( $\bar{x}$ ±s).” If the data do not follow a normal distribution, independent sample rank sum tests will be used for intergroup comparisons, and paired sample rank sum tests for intragroup comparisons, represented as [M (P25, P75)]. To investigate the impact of using reward strategies combined with neuromodulation techniques on upper limb dysfunction in chronic stroke survivors with low motivation over time, a repeated measure ANOVA will be performed, with rehabilitation motivation, motor function, functional independence, ICF activity, and participation as dependent variables. The model includes three groups (RStDCS group, RS group, and control group) and three test types (0, 3, and 6 weeks). Due to the strict requirements for repeated measurement data, missing cannot occur. If a sample lacks measurement data, all the data from that sample will be excluded from the model during analysis. A value of *p* less than 0.05, two-tailed, is considered statistically significant.

### 2.9. Ethics and dissemination

#### 2.9.1. Ethics statement

The study has received ethical from the Ethics Committee of Yueyang Hospital of Integrated Chinese and Western Medicine Affiliated to Shanghai University of Traditional Chinese Medicine (2021-122). All participating patients will provide written informed consent. Supplementary File 2 shows the consent form signed by participants. Trial registration number is ChiCTR2300069068.

Any adverse events reported by the subject or discovered by the investigator will be documented, and an analysis report of the incident process will also be recorded. Potential adverse events of Interest (AEIs) may include itching, redness, burning sensation on the skin, headache, tingling, fatigue, manic or hypomanic episodes, and seizures. All adverse events will be reported to the project manager, applicant, and clinical trial institution.

#### 2.9.2. Safety

In accordance with guidelines from the State Food and Drug Administration (2010), the Ethics Committee of Yueyang Hospital of Integrated Traditional Chinese and Western Medicine Affiliated to Shanghai University of Traditional Chinese Medicine will assign an independent staff member to conduct an annual review. This will include the experiment’s progress, the number of included subjects, the number of completed cases, the number of withdrawn cases, the management of serious adverse events, and any other events or new information that could affect the study.

Any amendments to the test protocol during the course of the study will be submitted to the Ethics Committee for review and approval prior to implementation. The Ethics Committee may request additional information related to the amendment review, including the contents and reasons for the amendment, its impact on the expected risks and benefits and how it affects the rights and safety of the subjects.

#### 2.9.3. Dissemination

Authorship will be determined according to the International Committee of Medical Journal Editors (ICMJE) guidelines. Results will be disseminated through peer-reviewed publications (preferably open access), networks of scientists, patient associations, professional and the public presentations, and relevant conferences. Study participants will receive regular updates about the progress and results through newsletters.

## 3. Discussion

In the process of outpatient, family, and social rehabilitation, promoting rehabilitation motivation can help establish the concept of “active health” among patients in an environment of limited medical resources. “Active health” concept refers to the ability to actively acquire sustainable health, have a healthy and fulfilling quality of life, and adapt socially. For stroke patients, active rehabilitation motivation is essential to establishing the concept of “active health.” As a rehabilitation medical team, it is our responsibility to increase patients’ awareness of active exercise and help them achieve rehabilitation goals. This involves providing scientific evaluation methods, teaching patients how to proactively identify problems, and adapting rehabilitation programs to implement “active health” concept. By doing so, we can reduce the economic and social burden on doctors, therapists, patients, and their families, while helping patients work toward the ultimate goal of rehabilitation: returning home and reintegrating into society.

This randomized controlled trial is designed to investigate the efficacy of RstDCS and RS in improving patients’ motivation, in comparison with conventional rehabilitation therapy, for 87 patients. The safety of RStDCS will be observed and its effects on motor function, ADL abilities, ICF activity, and social participation will be compared.

In this study, reward strategies treatment is based on social cognitive theory, self-determination theory, and economic behavior theory to intervene with stroke patients with low motivation.

The central goal of social cognitive theory is to enhance patients’ self-efficacy and self-regulation ability. Economic behavior theory aims to narrow down tempting distractions, helping patients focus on long-term goals. Self-determination theory seeks to support patients’ autonomy and stimulate intrinsic motivation.

By integrating these three theories, the reward training method encourages the patients to achieve their goals through the establishment of personalized, achievable yet challenging targets. This comprehensive approach breaks down large goals into smaller steps and combined with reward credits and behavioral feedback, helps reduce the decision-making bias of patients. This process supports patients in making decisions and behaviors consistent with their long-term goals, and visually helps them achieve these goals step by step. After 42 days of hospital and home reward training, when patients can see their improvement and progress after active exercising and completing daily small goals, the external motivation promoted by the reward strategy can transition into patients’ intrinsic motivation, effectively enhancing their drive for active exercise and rehabilitation.

This study acknowledges that low motivation for rehabilitation in chronic stroke patients is not only due to external environmental factors, but can also be a result of the neuropathic changes. About one-third of these patients exhibit functional decline in brain region related to processing motivation and associated network connectivity dysfunction. The dlPFC is believed to play an important role in computing the predicted capacity to successfully exert effort, enabling the dACC to compare the required work with potential benefits (46). Stimulation of the dlPFC with anodal tDCS can enhance the connectivity of motivation-related brain regions and their associated networks.

There are several limitations to consider. First, motivation is a dynamic process. In this study, although behavioral observation and other rating scales were used to evaluate patients’ motivation, there is a lack of assessment tools that could evaluate patients’ motivation level in real time. Brain imaging techniques such as functional near infrared spectroscopy (fNIRS), electroencephalogram (EEG), and magnetic resonance imaging (MRI) could be considered in the future. They can be used to detect motivation-related areas in the cerebral cortex and deep cerebral nuclei in real time. Second, the time cost of personalized printed pamphlets and posters for each patient is high, and it remains to be seen whether the digital version of an active exercise program can reduce the effort of therapists and doctors and have the same effect on patients. Third, whether the benefits of rehabilitation motivation enhanced by RStDCS can reduce the burden and cost of time and money for patients and their family’s needs to be verified with a larger sample size.

In conclusion, this study integrates social cognitive theory, economic behavioral science, rehabilitation medicine, and other related fields of knowledge. By using straightforward and feasible reward strategies (extrinsic reinforcement) combined with neural regulation techniques (intrinsic stimulation), we aim to improve the rehabilitation motivation of patients. This motivation will be observed through behavioral observation and examiner-rating scale. This approach offers an initial exploration pathway to improving patients’ rehabilitation motivation through a comprehensive strategy.

## Ethics statement

The studies involving human participants were reviewed and approved by the Ethics Committee of Yueyang Hospital of Integrated Chinese and Western Medicine Affiliated to Shanghai University of Traditional Chinese Medicine (2021-122). The patients/participants provided their written informed consent to participate in this study. Written informed consent was obtained from the individual(s) for the publication of any potentially identifiable images or data included in this article.

## Author contributions

PZ: proposal and protocol development, data acquisition, and trial coordination. WL: optimizing the laboratory protocols, data acquisition, and trial coordination. SC, JZ, and YC: protocol development and optimizing the research protocols. DX and XS: principal investigator. All authors contributed to the article and approved the submitted version.

## Funding

This project has been funded by the National Key R&D Program of China (Grant number: 2020YFC2004202). The project received joint support from Tongji University, Yueyang Hospital of Integrated Traditional Chinese and Western Medicine Affiliated to Shanghai University of Traditional Chinese Medicine, and Shuguang Hospital Affiliated to Shanghai University of Traditional Chinese Medicine. The project funders were not involved in data collection, analysis and interpretation of the data or manuscript writing.

## Conflict of interest

The authors declare that the research was conducted in the absence of any commercial or financial relationships that could be construed as a potential conflict of interest.

## Publisher’s note

All claims expressed in this article are solely those of the authors and do not necessarily represent those of their affiliated organizations, or those of the publisher, the editors and the reviewers. Any product that may be evaluated in this article, or claim that may be made by its manufacturer, is not guaranteed or endorsed by the publisher.
