# Supplementary material for: Modulated effectiveness of rehabilitation motivation by reward strategies combined with tDCS in stroke: study protocol for a randomized controlled trial
Source: Front Neurol. 2023 Jun 15;14:1200741. doi: 10.3389/fneur.2023.1200741 (PMC10310965; doi:10.3389/fneur.2023.1200741)
Supplement: Supplementary file 1 [file Data_Sheet_1.docx]

Supplementary Material

Modulated effectiveness of rehabilitation motivation by reward strategies combined with tDCS in stroke: study protocol for a randomized controlled trial.

**Ping Zhou^1,2†^**, **Wenxi Li^3†^**, **Jingwang Zhao^4^**, **Siyun Chen^4^, Yufeng Chen^4^, Xia Shen^1^*, Dongsheng Xu^2,4,5^***

*** Correspondence:**Dongsheng Xu and Xia Shen are co-corresponding authors for this paper.

Dongsheng Xu
[dxu0927@shutcm.edu.cn](mailto:dxu0927@shutcm.edu.cn)

Xia Shen

[christin417@hotmail.com](mailto:christin417@hotmail.com)

**Supplementary File 1:Rehabilitation Motivation Scale**

Rehabilitation Motivation Scale

|  | 1 | 2 | 3 | 4 |
| --- | --- | --- | --- | --- |
| Does the patient want to do rehabilitation exercises? | never | few | often | always |
| Does the patient complain of poor recovery? | never | few | often | always |
| Does the patient complain of excessive pain? | never | few | often | always |
| Will the patient try to do rehabilitation exercises? | never | few | often | always |
| Does the patient need too much encouragement? | never | few | often | always |
| Does the patient cooperate? | never | few | often | always |
| Does the patient find excuses to avoid recovery? | never | few | often | always |
| Does the patient ask or seek for resources? | never | few | often | always |

**Supplementary File 2: Consent Form Signed by participants**

Participant Consent and Signature

Clinical research project: Effectiveness of using reward strategies combined with transcranial direct current stimulation（RstDCS） on motivation in chronic stroke patients with upper limb disorders

Declaration of consent

-I have read the above introduction to this study, fully understand the full contents of the informed consent form, and have had the opportunity to discuss and ask questions about this study with doctors. All my questions were answered satisfactorily.

-I am aware of the risks and benefits that may arise from participating in this study. I understand that participation in the study is voluntary, I confirm that I have had sufficient time to consider it, and I understand that:

A I can always ask the doctor for more information.

B I can withdraw from the study at any time without discrimination or retaliation, and my medical treatment and rights will not be affected.

C If I need to take any other medication for a change in my condition, I will ask my doctor for advice beforehand or tell my doctor afterwards.

D I grant access to my research data to the Ethics Committee or the sponsor's representative.

E I consent □ or refuse □ to use my medical records for research other than this study.

F I will obtain a signed and dated copy of the informed consent. In the end, I decided to agree to participate in the study and try to follow the doctor's advice.

Date of subject's signature (handwritten)

Date:

Subject contact number：

Subject's legal guardian's signature (if necessary), (handwritten)

Date：

Guardian contact number：

Medical statement

I confirm that I have explained the details of the trial, including its powers and possible benefits and risks, and given the patient a signed copy of the informed consent.

The date of the investigator's (subject's informing) signature (handwritten)

Researcher contact number：

**Supplementary File 3:Active exercise program design library**

**of reward strategies treatment**

The Brunnstom staged treatment is as follows:

1、Treatment methods for stages I-II:

1. Maintain and improve joint mobility: Bobath handshake, roller training, etc. Use the healthy side to assist with passive movement on the affected side, perform lifting and forward extension arm movements.
2. Therapeutic activities for the upper limbs and hands:

During training, resistance patterns such as traction and compression are used to resist spasticity, use weight-bearing exercises, or perform activities under weighted conditions to reduce muscle spasms in the affected upper limb. Inhibit finger flexion training to reduce the degree of finger spasms. Separation movement training, such as holding a ball or a stick with the upper limbs.

1. Functional training for the upper limbs and hands

Training for upper limb motion control ability: Upper limb robot game training, upper limb holding ball training, and pushing a Bobath ball on the ground.

1. Daily life activity training
2. Encourage the affected hand to participate in daily activities, such as turning over and sitting up;
3. Wash the affected hand (three times a day, each time no less than 3 minutes);
4. Place the affected hand on the table and perform functional activities with the healthy side (three times a day, each time 15-20 minutes);
5. Add straight-arm support training for the affected limb during activities, such as standing and working at a high kitchen table (no less than 30 minutes per day);
6. Perform simple activities with both hands, such as holding a cloth to wipe the table (three times a day, each time 10 minutes);
7. Practice lifting the affected limb onto the thigh while sitting (two sets per day, 100 times per set);

g. Perform simple activities with both hands, such as holding a cup to drink water.

2、Treatment methods for stages II-IV:

A. Maintain and improve joint mobility: Bobath handshake, roller training, etc. Use the healthy side to assist with passive movement on the affected side, perform lifting and forward extension arm movements.

B. Therapeutic activities for the upper limbs and hands: during training, resistance patterns such as traction and compression are used to resist spasticity, use weight-bearing exercises, or perform activities under weighted conditions to reduce muscle spasms in the affected upper limb. Inhibit finger flexion training to reduce the degree of finger spasms. Separation movement training, such as holding a ball or a stick with the upper limbs.

C. Functional training for the upper limbs and hands

Training for upper limb motion control ability: upper limb robot game training, upper limb lifting device training, gymnastics stick training, etc. Coordination training for both hands: coordinated lifting and carrying of objects, wooden nail discs, jigsaw puzzles, and other activities.

D. Daily life activity training

a. Encourage the affected hand to participate in daily activities, such as turning over and sitting up;

b. Wash the affected hand (three times a day, each time no less than 3 minutes);

c. Place the affected hand on the table and perform functional activities with the healthy side (three times a day, each time 15-20 minutes);

d. Add straight-arm support training for the affected limb during activities, such as standing and working at a high kitchen table (no less than 30 minutes per day);

e. Perform simple activities with both hands, such as holding a cloth to wipe the table (three times a day, each time 10 minutes);

f. Practice lifting the affected limb onto the thigh while sitting (two sets per day, 100 times per set);

g. Perform simple activities with both hands, such as holding a cup to drink water.

3、Treatment methods for stages V-VI:

A. Maintain and improve joint mobility: actively move the affected upper limb, perform lifting and forward extension arm movements.

B. Therapeutic activities for the upper limbs and hands: training for shoulder abduction at 90° and elbow extension, palm rotation, gripping and releasing, wrist extension, and flexion-extension training for the paralyzed hand.

C. Functional training for the upper limbs and hands:

a. Coordination training for both hands: coordinated lifting and carrying of objects, wooden nail discs, jigsaw puzzles, and other activities;

b. Hand-eye coordination training: throwing and catching a ball, playing with a racket;

c. Finger grip and fine motor skills: board games, paper folding, flipping playing cards, knitting.

D. Daily life activity training

a. Use the affected limb to extract items in daily life more often;

b. Use the affected limb to hold cups and bowls while eating, peel bananas;

c. Hold combs, cups, etc. while grooming;

d. Button large buttons, put on socks while dressing;

e. Turn pages while reading, play cards during leisure time, fold paper, follow the rhythm of music to rotate sticks or hit castanets;

f. Rub and separate banknotes or open plastic bags with fingers while shopping;

g. Twist plastic bottle caps and cup lids, practice using spoons (three sets per day, each set for 30 minutes);

h. Use the affected hand to turn on/off lights, make phone calls;

i. Use the affected hand to twist open the toothpaste cap, use fingers to apply lipstick, etc.;

j. Use chopsticks with assistive devices for eating (suitable for right hemiplegia);

k. Use both hands when dressing.
